# Comparison of Continuous and Gait-synchronized Transcutaneous Electrical Nerve Stimulation During Gait Training in Chronic Stroke: A Pilot Randomized Trial

**DOI:** 10.31083/RN53766

**Published:** 2026-08-25

**Authors:** Ji-Min Na, Min-Keun Song, Hyeng-Kyu Park, In-Sung Choi, Junyeong Lee, Jungwon Yoon, Jae-Young Han

**Affiliations:** ^1^Department of Physical and Rehabilitation Medicine, Chonnam National University Medical School & Hospital, 61469 Jeonnam-Gwangju, Republic of Korea; ^2^Department of AI Convergence, Gwangju Institute of Science and Technology (GIST), 61005 Jeonnam-Gwangju, Republic of Korea

**Keywords:** gait, rehabilitation, stroke, transcutaneous electrical nerve stimulation, pilot projects

## Abstract

**Background:**

Post-stroke gait dysfunction may involve impaired integration of motor commands, somatosensory feedback, and phase-dependent peripheral sensory input during locomotion. Transcutaneous electrical nerve stimulation (TENS) combined with gait training may provide additional sensory input, but the relevance of different temporal stimulation profiles remains unclear.

**Objective:**

To evaluate the feasibility, safety, and preliminary clinical signals of continuous and gait-synchronized TENS during gait training in individuals with chronic stroke.

**Methods:**

Twenty-four participants with chronic stroke were randomized to continuous stimulation (CS), gait-synchronized stimulation (GS), or sham stimulation during standardized gait training. Participants received 12 intervention sessions over 4 weeks. CS delivered non-phase-specific stimulation, whereas GS delivered stimulation according to stance and swing phases. Outcomes were assessed at baseline, post-intervention, and 8-week follow-up. The primary longitudinal analysis was conducted in the modified intention-to-treat (mITT) population using baseline-adjusted linear mixed-effects models, with false discovery rate correction applied to group × time interaction *p*-values across estimable outcomes.

**Results:**

One participant withdrew before intervention without baseline assessment; therefore, 23 participants were included in the mITT analysis. Intervention adherence was high, with 99% of scheduled sessions completed, and no intervention-related adverse events were reported. Comfortable gait speed showed no significant group × time interaction. Maximal gait speed and Berg Balance Scale showed nominal group × time interaction effects before correction, but these did not remain significant after false discovery rate correction. No robust group × time interaction effects were observed after correction. Exploratory per-protocol analyses were unadjusted for baseline imbalance and were interpreted as supportive only.

**Conclusions:**

Continuous and gait-synchronized TENS combined with standardized gait training were feasible and well tolerated in individuals with chronic stroke. The adjusted analysis did not provide robust evidence of differential clinical effects among stimulation-timing groups after correction for multiple outcome testing. Larger, adequately powered trials are needed to determine whether continuous or gait-synchronized TENS provides clinically meaningful benefits during post-stroke gait rehabilitation.

**Clinical Trial Registration:**

No: KCT0008481. Registered 17 May, 2023, https://cris.nih.go.kr/cris/search/detailSearch.do?seq=24719&search_page=L.

## 1. Introduction

Post-stroke gait dysfunction reflects impairment in distributed neural systems involved in sensorimotor control, rather than an isolated impairment of lower-limb movement. Human walking requires the integration of descending motor commands, spinal locomotor circuitry, proprioceptive and cutaneous afferent input, and task-dependent postural adjustments. After stroke, disruption of motor and sensory networks may alter the processing and integration of sensory feedback, contributing to impaired inter-limb coordination, reduced dynamic balance, and inefficient locomotor adaptation. Accordingly, chronic post-stroke gait impairment can be understood as a disorder of brain–body interaction in which altered sensory processing and residual motor capacity shape locomotor performance.

Sensory input is particularly important during walking, because lower-limb afferent signals provide phase-dependent information about limb position, loading, foot contact, and movement progression. These signals interact with ongoing motor commands and contribute to stance stability, swing initiation, limb advancement, and postural control. In individuals with chronic stroke, gait is often slow, asymmetric, and unstable, even when independent ambulation is regained. These clinical manifestations may reflect, at least in part, impaired sensorimotor integration during locomotion. This provides a rationale for approaches that incorporate peripheral sensory input into locomotor training, with the clinical goal of improving gait and balance as a downstream implication.

Task-specific gait training is widely used; however, approaches that provide or augment sensory input during locomotor practice may support sensorimotor activation and locomotor-related function [1,2]. Recent evidence supports the role of sensory and proprioceptive input modulation in improving gait and balance in individuals with chronic stroke [3]. These findings suggest that augmenting sensory input during movement may influence post-stroke locomotor performance, although the optimal temporal structure for delivering such input remains uncertain.

Transcutaneous electrical nerve stimulation (TENS), traditionally used for analgesia, has increasingly been investigated in stroke rehabilitation, with randomized studies and systematic reviews reporting potential benefits for balance, mobility, walking capacity, spasticity, and lower-limb motor function [4,5,6,7,8]. However, findings remain variable, possibly reflecting differences in stimulation parameters such as intensity, electrode placement, and timing relative to movement.

In the present study, TENS was conceptualized as a method for providing externally generated peripheral sensory input during gait training, allowing the temporal structure of sensory input to be varied. Continuous stimulation and gait-synchronized stimulation represent distinct temporal sensory-input profiles: the former provides non-phase-specific input throughout walking, whereas the latter provides input aligned with specific gait phases. Because sensory inputs and motor demands influence gait-related control [9], and locomotion involves phase-specific neuromuscular control patterns, aligning stimulation with discrete gait events may provide temporally specific sensory input during functionally relevant phases of gait [10]. Wearable sensing technologies enable real-time gait phase detection [11], allowing stimulation to be linked to specific phases of the gait cycle. However, the clinical and neurophysiological relevance of gait-synchronized TENS during post-stroke gait training remains limited, and direct comparisons between continuous and gait-synchronized stimulation during gait training are lacking.

Individuals with chronic stroke may retain some capacity for locomotor adaptation [12,13,14], suggesting that the temporal structure of sensory input during gait training may be relevant to locomotor-related functional change. However, whether such temporally structured stimulation is associated with different preliminary outcome patterns compared with continuous stimulation remains unclear.

The aim of this pilot randomized clinical trial was to evaluate the feasibility, safety, and preliminary clinical signals of continuous versus gait-synchronized TENS during gait training in individuals with chronic stroke. We examined whether different temporal patterns of peripheral sensory input during gait training were associated with different preliminary locomotor-related outcome patterns, hypothesizing that stimulation timing may be associated with different functional trajectories.

## 2. Materials and Methods

### 2.1 Study Design

This study was a randomized, single-center, assessor-blinded pilot clinical trial designed to evaluate the feasibility, safety, and preliminary outcome changes of TENS combined with gait training in individuals with chronic stroke. The study was conducted in the Department of Physical and Rehabilitation Medicine at Chonnam National University Hospital, Gwangju, Republic of Korea. The completed CONSORT checklist is provided in the **Supplementary Material**.

### 2.2 Participants

Participants with chronic stroke were recruited from the outpatient rehabilitation setting of the Department of Physical and Rehabilitation Medicine at Chonnam National University Hospital between June 2023 and December 2023. Recruitment was completed after the target sample size had been reached, and follow-up assessments were completed in February 2024.

No formal efficacy-based sample size calculation was performed because this was a pilot randomized trial designed primarily to evaluate feasibility, safety, implementation, and preliminary clinical signals rather than to provide confirmatory evidence of treatment efficacy. Methodological guidance for pilot and feasibility studies emphasizes that such studies are generally intended to assess trial procedures, recruitment feasibility, intervention delivery, and variability estimates for future definitive trials, rather than to test efficacy hypotheses [15]. Several pragmatic or rule-of-thumb approaches for pilot trial sample size planning have been proposed [16,17]. Therefore, the target sample size was set at 24 participants, with eight participants per group. This target was determined pragmatically based on expected recruitment capacity, available resources, participant burden, and the practical constraints of delivering the 4-week device-based gait-training intervention. This sample size was not intended to provide precise effect estimates or sufficient statistical power to detect between-group treatment effects.

Patients were included if they met all of the following criteria: (1) age between 20 and 90 years; (2) diagnosis of ischemic or hemorrhagic stroke with onset more than 6 months before enrollment; (3) presence of hemiplegia; (4) Functional Ambulation Category (FAC) score between 2 and 4; (5) ability to understand and follow instructions required for gait training and study assessments; and (6) provision of written informed consent.

Exclusion criteria were as follows: (1) gait or balance impairment due to other neurodegenerative, neuromuscular, or musculoskeletal conditions; (2) skin lesions or vascular conditions at electrode attachment sites, such as peripheral arterial disease; (3) unstable vital signs; (4) active malignant disease; (5) inability to follow the study protocol for any other reasons; or (6) contraindications to TENS.

### 2.3 Randomization and Blinding

After written informed consent was obtained and eligibility was confirmed, participants were assigned a study identification number and randomly allocated to one of three groups: continuous stimulation (CS), gait-synchronized stimulation (GS), or sham stimulation. A computer-generated randomization sequence was created using Random Allocation Software 2.0 (Mahmood Saghaei, Isfahan, Iran). Simple randomization was performed with a 1:1:1 allocation ratio.

Allocation concealment was maintained using sequentially numbered, opaque, sealed envelopes. The random allocation sequence was generated by an independent researcher who was not involved in participant recruitment, intervention delivery, or outcome assessment. Participants were enrolled by the study investigators, and group assignments were determined by opening the sealed envelopes after enrollment.

This study used an assessor-blinded design. Because of the nature of the intervention, therapists could not be blinded to group allocation, and participant blinding may not have been fully maintained. Outcome assessors were not involved in intervention delivery and remained blinded to group allocation throughout the study. Separate case report forms were used for outcome assessment, and assessors did not have access to allocation information.

### 2.4 Interventions

#### 2.4.1 Experimental setup and TENS protocols

The intervention system consisted of a low-frequency electrical stimulator (FIT 3.0, Compex, Guildford, UK) and a microcontroller unit (myRIO, National Instruments Inc., Austin, TX, USA), which were attached to a waist belt worn by each participant. The microcontroller operated custom software developed using LabVIEW 2017 (National Instruments Inc.) and controlled the timing of TENS delivery at a sampling rate of 100 Hz.

Gait phases were detected using a pair of instrumented insoles equipped with force-sensing resistor (FSR) sensors (A401-25, Tekscan, Norwood, MA, USA) connected to the microcontroller module. Four FSR sensors were positioned under the heel, toe, first metatarsal, and fifth metatarsal regions. Ground contact was identified using predefined threshold values, enabling detection of stance and swing phases during gait [18]. Before training, the insole sensors were calibrated with the participant seated and both feet off the ground. The stance phase was detected when the summed load measured by the four FSR sensors exceeded 2 kg, whereas the swing phase was identified when the summed load fell below this threshold.

To evaluate the technical validity of the gait-phase detection algorithm, the FSR-based insole system was compared with a commercial wearable inertial measurement unit (IMU)-based motion analysis system (myoMOTION, Noraxon, Scottsdale, AZ, USA; accuracy: ±1° in the vertical plane and ±2° in the horizontal plane). Gait-phase events were calculated using MR 3.16 software (Noraxon). In a validation test involving 200 overground walking steps from a healthy young adult, the FSR-based insole system detected 198 of 200 foot-contact events, corresponding to a foot-contact event detection rate of 99% and a missed-event rate of 1%. The mean phase delay was 25.62 ± 14.06 ms for stance-phase detection and 28.20 ± 12.89 ms for swing-phase detection. The validation procedure is shown in **Supplementary Fig. 1**. These data were used to document the detection rate, missed-event rate, phase delay, and consistency of the gait-phase detection algorithm before application of the gait-synchronized stimulation protocol.

TENS electrodes were placed over the tibialis anterior and gastrocnemius muscles on the hemiplegic side. Before the intervention, the sensory threshold of the affected limb was determined for each participant. Stimulation intensity was set at 1.5–2 times the individual sensory threshold without visible muscle contraction. For participants who did not perceive the stimulation, the intensity was briefly increased until visible muscle contraction was observed and was then reduced to a level at which no muscle contraction occurred.

TENS was delivered at a frequency of 100 Hz with a pulse width of 0.2 ms [5,19]. Each participant was scheduled to receive 12 intervention sessions over 4 weeks, with three sessions per week. TENS was applied for 20 minutes during each gait-training session according to the assigned stimulation-timing protocol.

**Fig. 1.**
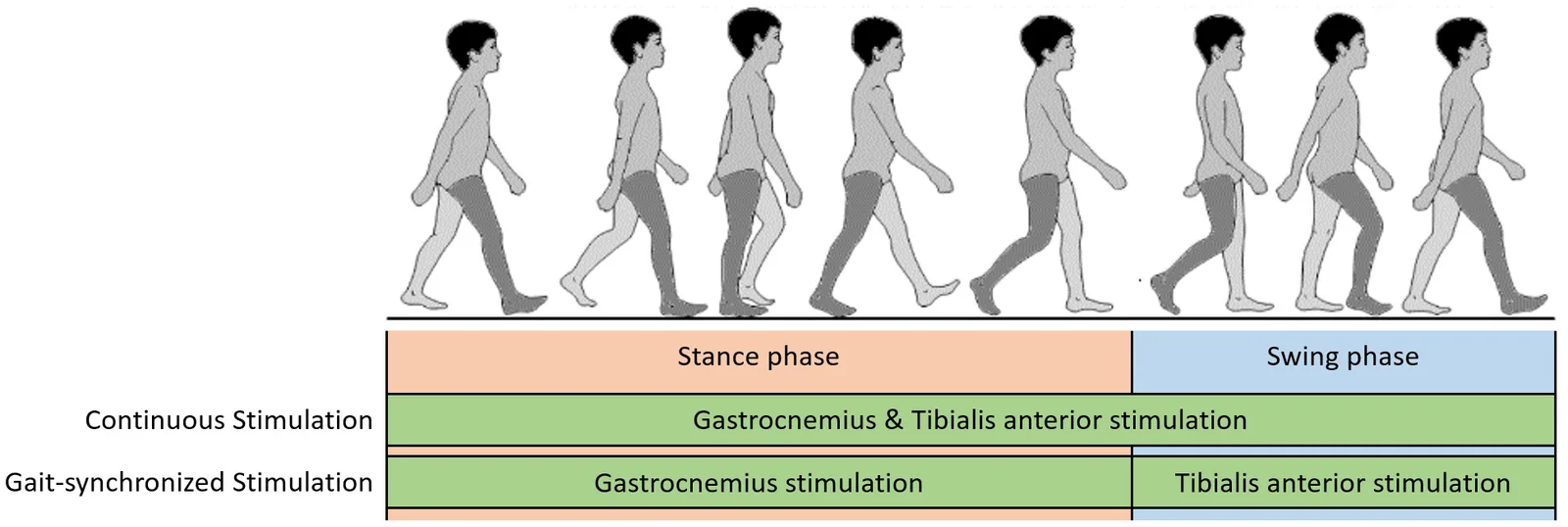
**Comparison of continuous and gait-synchronized stimulation protocols**. Schematic representation of the gait cycle showing the application of continuous and gait-synchronized stimulation. In the continuous protocol, both the gastrocnemius and tibialis anterior muscles are stimulated throughout the gait cycle. In the gait-synchronized protocol, gastrocnemius stimulation is applied during the stance phase, while tibialis anterior stimulation is applied during the swing phase. The gait cycle illustration is adapted from “Phases of gait cycle” (Physiopedia), licensed under CC BY-SA 4.0. Modifications were made for layout and clarity.

In the CS group, TENS was delivered continuously through both stimulation channels throughout the gait training session. In the GS group, TENS was delivered according to the gait phase: stimulation was applied to the anterior lower leg during the swing phase and to the posterior lower leg during the stance phase. In the sham stimulation group, stimulation was delivered to both channels for the initial 15 seconds and was then discontinued. Stimulation or gait training was discontinued if the participant requested cessation or if the supervising therapist judged that continuation was inappropriate for safety reasons. An emergency stop button was available during the intervention. The stimulation protocols are illustrated in Fig. 1.

#### 2.4.2 Gait Training

All participants received standardized gait training for post-stroke hemiplegia in the physical therapy unit of Chonnam National University Hospital. Each gait-training session lasted 20 minutes. The session consisted of two 10-minute stages: a basic gait-training stage, including level-ground walking, followed by an advanced gait-training stage, including stair climbing, sideways walking, and backward walking. If a participant was unable to perform the advanced tasks because of functional limitations, the basic gait-training stage was continued or repeated so that the total gait-training duration remained 20 minutes.

All training sessions were conducted under the supervision or assistance of a licensed physical therapist. Assistive devices were used as needed according to each participant’s functional status. Intervention adherence was calculated as the proportion of completed sessions among the scheduled sessions and was summarized descriptively.

### 2.5 Outcome Assessment

**Fig. 2.**
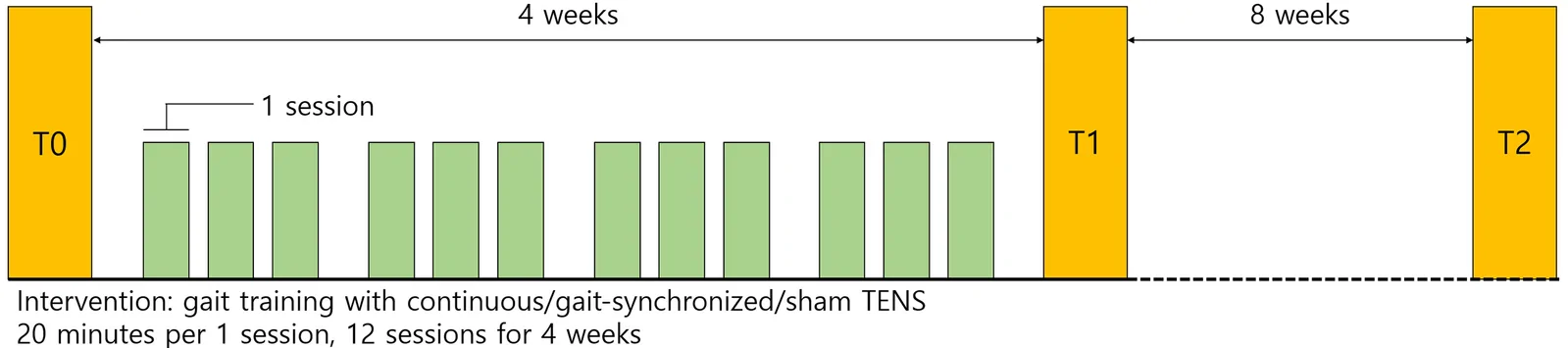
**Study timeline and intervention schedule**. Participants received 12 sessions of continuous, gait-synchronized, or sham transcutaneous electrical nerve stimulation (TENS) during gait training (3 sessions per week for 4 weeks). Outcome measures were assessed at baseline (T0), immediately after the intervention (T1), and 8 weeks post-intervention (T2).

The assessment timeline is shown in Fig. 2. All outcomes were assessed at baseline (T0), immediately after the 4-week intervention (T1), and at the 8-week follow-up (T2) by blinded assessors.

The primary outcomes were comfortable and maximal gait speeds measured using the 10-meter walk test (10MWT) with a flying-start protocol. The 10MWT was selected because of its established validity and responsiveness for evaluating gait performance in individuals with stroke [20]. Secondary outcomes included the Timed Up and Go (TUG) test and the Berg Balance Scale (BBS), which are validated measures of functional mobility and balance in stroke populations [21,22]. Additional outcomes included manual muscle testing (MMT) of the hip, knee, and ankle on both the ipsilesional and contralesional sides, the FAC, the Korean version of the Modified Barthel Index (K-MBI), and health-related quality of life assessed using the Korean version of the EuroQol-5 Dimension-3 Level (EQ-5D-3L). The five EQ-5D-3L descriptive dimension scores were summed, with higher scores indicating more problems.

Adverse events were monitored throughout the intervention period, including skin irritation, discomfort, pain, fatigue, falls, abnormal vital signs, and any other intervention-related events.

### 2.6 Statistical Analysis

Descriptive statistics and baseline comparisons were performed using SPSS Statistics for Windows, version 27.0.0.0 (IBM Corp., Armonk, NY, USA). Baseline-adjusted longitudinal analyses were performed using R software, version 4.6.0 (R Foundation for Statistical Computing, Vienna, Austria). Continuous variables are presented as mean ± standard deviation, and categorical variables are presented as number or number/number, as appropriate. Baseline characteristics were compared among the three groups using one-way analysis of variance for continuous variables and Fisher’s exact test for categorical variables.

The primary longitudinal analysis was conducted in the modified intention-to-treat (mITT) population. Of the 24 randomized participants, one participant withdrew before the initiation of the intervention and did not undergo baseline assessment; therefore, this participant was excluded from the mITT analysis because no baseline or post-baseline outcome data were available. The mITT population included 23 participants: 8 in the continuous stimulation group, 7 in the gait-synchronized stimulation group, and 8 in the sham stimulation group.

Clinical outcomes were analyzed using baseline-adjusted repeated-measures analysis of covariance implemented as a linear mixed-effects model. For each outcome, post-baseline values at T1 and T2 were used as repeated dependent variables, and the corresponding baseline value at T0 was included as a covariate. The model included group, time, group × time interaction, and baseline value as fixed effects, with a participant-specific random intercept. Type III F-tests were used, and denominator degrees of freedom were estimated using Satterthwaite’s approximation. Missing outcome data were not imputed; available observations were used in the mixed-effects model.

Estimated marginal means with 95% confidence intervals were calculated for each group at each post-baseline time point. Pairwise between-group comparisons were performed using Tukey-adjusted *p*-values. Benjamini–Hochberg false discovery rate correction was applied to the group × time interaction *p*-values across all estimable outcomes. Statistical significance was defined as a two-sided *p*-value < 0.05, with false discovery rate (FDR)-adjusted *p*-values used to interpret multiple outcome testing.

An exploratory per-protocol analysis was additionally performed after excluding two participants with eligibility violations. The per-protocol population included 21 participants: 8 in the continuous stimulation group, 6 in the gait-synchronized stimulation group, and 7 in the sham stimulation group. Repeated-measures analysis of variance was conducted in this population, with effect sizes reported as partial eta squared (ηp^2^), and the results are presented in the **Supplementary Material**.

## 3. Results

### 3.1 Participant Flow and Baseline Characteristics

**Fig. 3.**
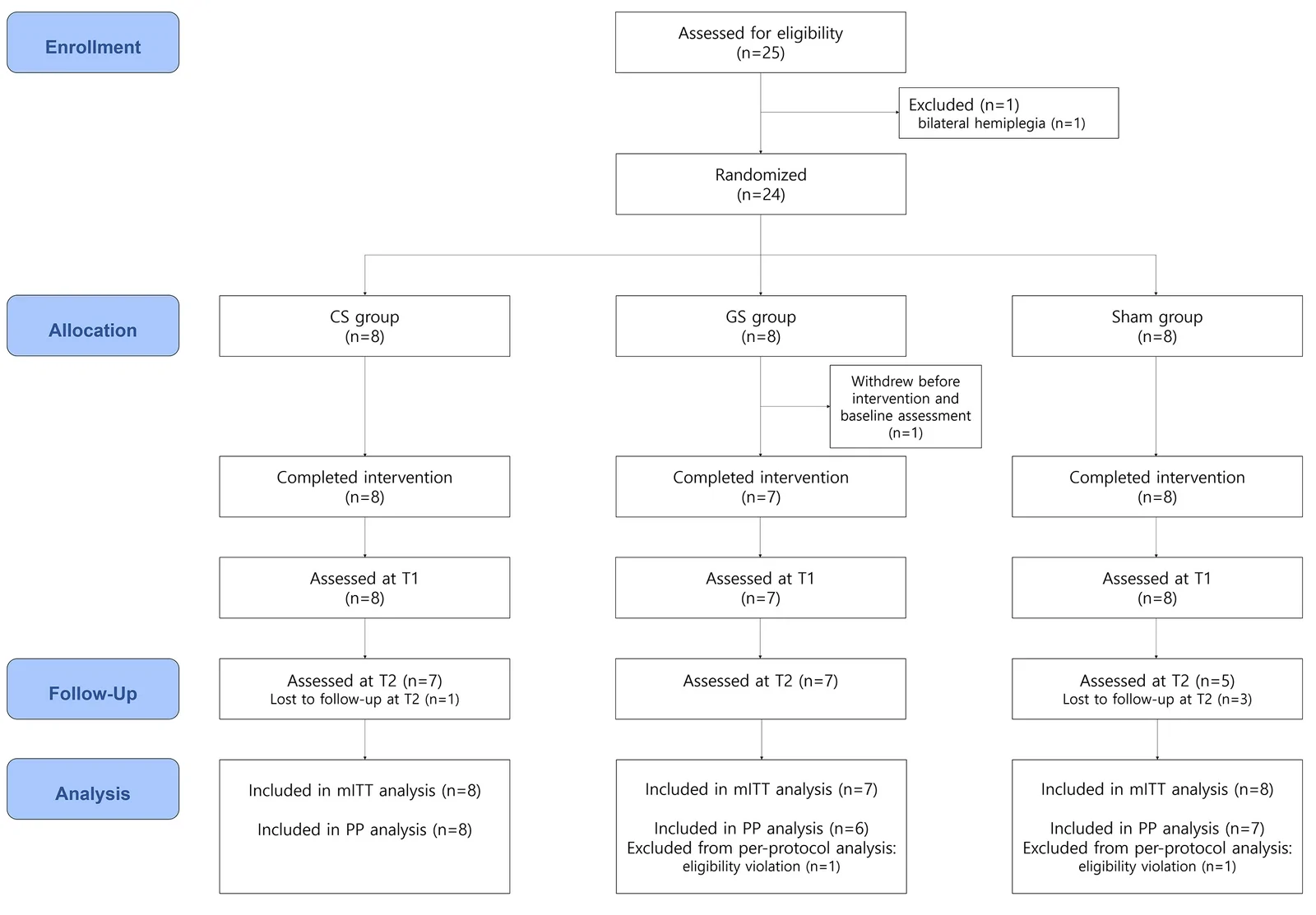
**Consolidated Standards of Reporting Trials (CONSORT) flow diagram of the study**. Outcomes were assessed immediately after the 4-week intervention (T1) and at the 8-week follow-up (T2). The modified intention-to-treat population was used for the primary longitudinal analysis, and the per-protocol population was used for exploratory analysis. Abbreviations: mITT, modified intention-to-treat; PP, per-protocol; CS, continuous stimulation; GS, gait-synchronized stimulation.

A total of 25 participants were assessed for eligibility, of whom one was excluded because of bilateral hemiplegia. The remaining 24 participants were randomized to the CS, GS, or sham groups. One participant in the GS group withdrew consent before the intervention and did not undergo baseline assessment; therefore, 23 participants were included in the mITT population: 8 in the CS group, 7 in the GS group, and 8 in the sham group. During data verification, two participants, one in the GS group and one in the sham group, were found to have eligibility violations because their FAC score was 1. These participants were therefore excluded from the per-protocol analysis. The per-protocol population therefore included 21 participants: 8 in the CS group, 6 in the GS group, and 7 in the sham group (Fig. 3).

Baseline demographic and clinical characteristics of the mITT population are summarized in Table 1. No statistically significant between-group differences were observed in age, side of hemiplegia, or comorbidities (all *p* > 0.05). However, several functional measures differed among groups at baseline. Comfortable and maximal gait speeds were higher in the CS group than in the GS and sham groups. Significant between-group differences were also observed for the TUG and BBS. FAC, K-MBI, and EQ-5D-3L sum score did not differ significantly among groups at baseline.

**Table 1.** **Baseline characteristics of the modified intention-to-treat population**.

Variables		CS group (n = 8)	GS group (n = 7)	Sham group (n = 8)	*p*-value
Age (years)		63.1 ± 11.5	73.6 ± 4.0	62.8 ± 14.8	0.141
Height (cm)		162.6 ± 5.7	162.6 ± 5.7	166.9 ± 6.2	0.274
Body weight (kg)		64.4 ± 8.2	66.0 ± 5.3	68.3 ± 14.4	0.748
Sex, n (male/female)		4/4	5/2	5/3	0.866
Side of hemiplegia, n (right/left)		6/2	3/4	5/3	0.496
Hypertension, n		5	2	4	0.508
Diabetes mellitus, n		2	2	2	1.000
Dyslipidemia, n		1	1	3	0.557
Cardiac disease, n		2	2	0	0.406
10-meter walk test (m/s)					
	Comfortable speed	1.0 ± 0.2	0.6 ± 0.2	0.6 ± 0.3	0.004*
	Maximal speed	1.2 ± 0.2	0.7 ± 0.3	0.7 ± 0.4	0.009*
Timed Up and Go test (s)		7.2 ± 1.2	11.9 ± 4.8	16.2 ± 9.7	0.033*
Berg Balance Scale (points)		45.1 ± 5.4	30.1 ± 7.8	30.0 ± 7.8	<0.001*
Functional Ambulation Category (level)		4.5 ± 0.5	3.4 ± 1.1	3.4 ± 1.2	0.061
Korean version of the Modified Barthel Index (points)		87.9 ± 5.7	79.3 ± 13.1	74.2 ± 18.9	0.159
EQ-5D-3L (sum score)		7.2 ± 2.3	8.6 ± 1.9	8.6 ± 1.7	0.312

Values are presented as mean ± standard deviation for numerical variables and as number or number/number for categorical variables, as appropriate. Sex is presented as male/female, and side of hemiplegia is presented as right/left. Between-group comparisons were performed using one-way analysis of variance for numerical variables and Fisher’s exact test for categorical variables. *p < 0.05. Higher EQ-5D-3L sum scores indicate more problems. Abbreviations: EQ-5D-3L, EuroQol-5 Dimension-3 Level.

### 3.2 Feasibility and Safety

Intervention adherence was high, with 99% of scheduled sessions completed (274/276 sessions). No participants discontinued the intervention because of stimulation-related discomfort. No intervention-related adverse events were reported during the study period. One participant reported facial flushing during the trial, which was judged to be unrelated to the intervention.

### 3.3 Preliminary Clinical Outcomes

The primary longitudinal analysis was conducted in the mITT population using baseline-adjusted linear mixed-effects models (Table 2).

**Table 2.** **Baseline-adjusted linear mixed-effects model analysis of post-intervention and follow-up outcomes in the modified intention-to-treat population**.

Variables		Model term	F	df1	df2	*p*-value	FDR-adjusted *p*-value
**Primary outcomes**							
10-meter walk test (m/s)							
	Comfortable speed	Group	0.265	2	21.84	0.770	-
		Time	11.405	1	18.43	0.003*	-
		Group × Time	1.933	2	18.39	0.173	0.173
	Maximal speed	Group	1.968	2	24.11	0.162	-
		Time	10.750	1	20.94	0.004*	-
		Group × Time	3.531	2	20.87	0.048*	0.123
**Secondary outcomes**							
	Timed Up and Go test (s)	Group	2.396	2	22.90	0.114	-
		Time	0.394	1	19.03	0.537	-
		Group × Time	2.131	2	18.99	0.146	0.173
	Berg Balance Scale (points)	Group	0.024	2	22.53	0.976	-
		Time	10.729	1	19.99	0.004*	-
		Group × Time	4.685	2	19.89	0.022*	0.123
	Functional Ambulation Category (level)	Group	0.027	2	42	0.974	-
		Time	1.131	1	42	0.294	-
		Group × Time	2.981	2	42	0.062	0.123
	Korean version of the Modified Barthel Index (points)	Group	-	-	-	-	-
		Time	-	-	-	-	-
		Group × Time	-	-	-	-	-
	EQ-5D-3L (sum score)	Group	7.752	2	21.08	0.003*	-
		Time	2.329	1	19.16	0.143	-
		Group × Time	2.549	2	19.10	0.104	0.157

Baseline-adjusted linear mixed-effects models were fitted separately for each outcome. Post-intervention and follow-up values were modeled as repeated outcomes. Fixed effects included group, time, group × time interaction, and the corresponding baseline value. Participant ID was included as a random intercept. Fixed effects were evaluated using F-tests with Satterthwaite approximation for denominator degrees of freedom. Missing post-baseline outcome data were not imputed; the mixed-effects models used all available post-baseline observations. False discovery rate-adjusted *p*-values were calculated using the Benjamini–Hochberg procedure for the group × time interaction terms across the six prespecified main clinical outcomes; MMT outcomes were treated as a separate exploratory family. K-MBI was not included in the baseline-adjusted mixed-effects model because post-baseline values showed insufficient variability for reliable model estimation. Higher EQ-5D-3L sum scores indicate more problems. Unadjusted *p*-values are reported descriptively, whereas FDR-adjusted *p*-values were used for interpreting group × time interactions across outcomes. *p < 0.05. Abbreviations: df, degrees of freedom; FDR, false discovery rate; K-MBI, Korean version of the Modified Barthel Index.

For comfortable gait speed, no significant group × time interaction was observed. A significant main effect of time was observed, indicating an overall change in comfortable gait speed across post-baseline assessments after adjustment for baseline values. For maximal gait speed, the group × time interaction was significant before correction for multiple outcome testing but was not significant after false discovery rate correction.

Among secondary outcomes, the group × time interaction for BBS was significant before correction for multiple outcome testing but was not significant after false discovery rate correction. No significant group × time interactions were observed for the TUG test, Functional Ambulation Category, or EQ-5D-3L after baseline adjustment. Significant main effects of time were observed for BBS, indicating overall post-baseline change over time after adjustment for baseline values. The K-MBI model was not estimable because post-baseline values showed insufficient variability for reliable model estimation. No significant group × time interactions were observed for MMT across the assessed lower-limb muscle groups after FDR correction (**Supplementary Table 1**). Exploratory baseline-adjusted pairwise comparisons between groups at each post-baseline time point are presented in **Supplementary Table 2**.

The exploratory per-protocol repeated-measures analysis of variance (ANOVA) results are presented in **Supplementary Table 3**. Several group × time interaction effects reached nominal statistical significance in the per-protocol analysis; however, these analyses were not adjusted for baseline imbalance and were performed in the smaller per-protocol population. Therefore, they were interpreted as supportive and exploratory.

Baseline-adjusted estimated marginal means with 95% confidence intervals for each group at T1 and T2 are presented in Table 3. Descriptively, the adjusted means suggested greater numerical increases from T1 to T2 in maximal gait speed in the CS group and in BBS in the GS group. However, these between-group temporal patterns did not remain statistically significant after correction for multiple outcome testing.

**Table 3.** **Baseline-adjusted estimated marginal means of post-intervention and follow-up outcomes in the modified intention-to-treat population**.

Variables	Time	CS adjusted mean (95% CI)	GS adjusted mean (95% CI)	Sham adjusted mean (95% CI)
Comfortable speed (m/s)	T1	0.727 (0.643 to 0.812)	0.686 (0.607 to 0.764)	0.736 (0.661 to 0.811)
	T2	0.790 (0.704 to 0.876)	0.756 (0.678 to 0.834)	0.742 (0.660 to 0.825)
Maximal speed (m/s)	T1	0.893 (0.821 to 0.965)	0.849 (0.780 to 0.917)	0.889 (0.823 to 0.955)
	T2	0.994 (0.920 to 1.068)	0.868 (0.799 to 0.936)	0.912 (0.837 to 0.988)
Timed Up and Go test (s)	T1	11.391 (9.830 to 12.952)	12.606 (11.086 to 14.125)	9.963 (8.426 to 11.500)
	T2	10.844 (9.250 to 12.438)	11.972 (10.453 to 13.492)	10.629 (8.925 to 12.334)
Berg Balance Scale (points)	T1	37.257 (34.610 to 39.905)	35.922 (33.524 to 38.319)	37.740 (35.465 to 40.015)
	T2	38.521 (35.773 to 41.269)	40.207 (37.810 to 42.605)	37.845 (35.170 to 40.520)
Functional Ambulation Category (level)	T1	3.794 (3.530 to 4.059)	3.781 (3.510 to 4.053)	4.031 (3.775 to 4.286)
	T2	4.079 (3.799 to 4.360)	4.067 (3.796 to 4.338)	3.781 (3.453 to 4.109)
EQ-5D-3L (sum score)	T1	7.397 (6.642 to 8.151)	8.059 (7.263 to 8.856)	8.595 (7.847 to 9.342)
	T2	7.704 (6.890 to 8.518)	7.774 (6.977 to 8.570)	9.696 (8.766 to 10.626)

Values are estimated marginal means with 95% confidence intervals derived from the baseline-adjusted linear mixed-effects models. Estimated marginal means were adjusted for the corresponding baseline value and calculated at the overall mean baseline value for each outcome. Statistical test results are presented in Table 2. Estimated marginal means are not presented for K-MBI because the corresponding mixed-effects model was not estimable due to insufficient post-baseline variability. Higher EQ-5D-3L sum scores indicate more problems. Abbreviations: CI, confidence interval; T1, post-intervention; T2, follow-up.

## 4. Discussion

### 4.1 Principal Findings

This pilot randomized trial evaluated the feasibility, safety, and preliminary clinical signals of TENS combined with standardized gait training in individuals with chronic stroke. The main findings were that both CS and GS could be implemented with high adherence, without stimulation-related discontinuation or intervention-related adverse events. These findings support the feasibility and safety of delivering peripheral sensory stimulation during supervised gait training in this population.

The clinical outcome findings should be interpreted cautiously, because baseline imbalance was present in several functional measures. The baseline-adjusted mITT analysis did not provide robust evidence of differential longitudinal effects among the stimulation-timing groups after correction for multiple outcome testing. Although some nominal signals were observed in gait- and balance-related outcomes, these findings should be interpreted as preliminary and hypothesis-generating rather than as evidence of treatment efficacy.

Exploratory per-protocol findings are provided in the **Supplementary Material**. These results may be useful for identifying potential outcome domains for future studies, but they were based on a smaller population and were not adjusted for baseline imbalance. Therefore, they should be considered supportive rather than confirmatory.

### 4.2 Interpretation in Relation To Sensory Input, Sensorimotor Integration, and Gait Training

TENS applied during gait training may provide additional peripheral sensory input during repeated task-oriented locomotor practice. Task-oriented training is a widely used approach in stroke rehabilitation [23], and sensory input training has been investigated as a strategy to support motor function recovery after stroke [24]. During walking, sensory input may be particularly relevant because gait requires continuous coordination among motor commands, somatosensory feedback, postural control, and environmental demands. Locomotor rehabilitation also relies in part on experience-dependent plasticity and motor learning processes [25,26], and some capacity for locomotor adaptation may be retained even in the chronic stage after stroke [12,13,14].

In this study, stimulation was delivered during standardized gait training rather than as an isolated sensory intervention. Therefore, the potential relevance of TENS should be interpreted in the context of active, task-specific locomotor practice. Because the present study did not directly measure afferent transmission, cortical excitability, proprioceptive function, or motor learning, the current findings cannot establish a neural mechanism. However, they provide a feasibility- and hypothesis-generating basis for further investigation of how peripheral sensory input delivered during gait training may interact with post-stroke locomotor rehabilitation.

### 4.3 Continuous and Gait-Synchronized TENS as Distinct Temporal Sensory-Input Profiles

The CS and GS protocols should be interpreted as distinct temporal sensory-input profiles rather than as competing protocols in which one is superior to the other. CS provided non-phase-specific sensory input throughout gait training, whereas GS provided phase-linked input to the anterior lower leg during swing and to the posterior lower leg during stance. Because gait consists of stance and swing phases with different mechanical and sensory demands [27], temporally aligned stimulation may provide sensory input that coincides with phase-specific motor activity. Gait-synchronized stimulation approaches have been investigated in post-stroke gait rehabilitation [6,10,28], but direct comparison with continuous TENS during gait training remains limited.

The adjusted mITT findings did not demonstrate that either stimulation-timing protocol was superior to sham stimulation or to the other active protocol. Because direct neurophysiological measures were not obtained and corrected clinical effects were not robust, the present trial cannot determine whether either temporal profile altered sensorimotor integration, afferent processing, or motor adaptation.

### 4.4 Clinical and Translational Implications

From a translational perspective, the feasibility and safety findings suggest that both continuous and gait-synchronized TENS can be incorporated into supervised gait training without major implementation barriers in this pilot setting. Similar feasibility-oriented approaches have been explored with other non-invasive stimulation modalities delivered during gait training in individuals with chronic stroke, although the stimulation modality and target mechanisms differ from those of TENS [29]. A novel aspect of this study is the direct comparison of continuous and gait-synchronized TENS delivered during overground gait training using a wearable real-time gait-phase detection system. Accordingly, this study provides not only preliminary clinical observations but also practical technical implementation data on wearable synchronized stimulation during supervised post-stroke gait rehabilitation.

The clinical outcome findings should not be interpreted as sufficient to change clinical practice. After adjustment for baseline values and correction for multiple outcome testing, the study did not provide robust evidence of differential longitudinal effects among the stimulation-timing groups and does not establish efficacy, superiority, or an optimal stimulation-timing strategy. Rather, the findings demonstrate that both continuous and gait-synchronized TENS can be safely integrated into gait training using a wearable real-time stimulation system and provide preliminary clinical signals to inform the design of future adequately powered randomized trials.

The nominal signals observed in gait- and balance-related outcomes may help inform future trial planning because walking speed, functional mobility, and balance are clinically relevant domains in stroke rehabilitation [20,21,22,30]. These findings are also consistent with broader rehabilitation interest in somatosensory stimulation and proprioceptive feedback-based approaches for improving gait and balance after stroke [31,32]. Larger confirmatory trials are needed to determine whether continuous or gait-synchronized TENS provides clinically meaningful benefits beyond standardized gait training and sham stimulation.

### 4.5 Limitations

This study has several limitations. First, it was a pilot trial with a small sample size and was conducted at a single center, which limits statistical power, precision of effect estimates, and generalizability. The study was not designed or powered to provide confirmatory evidence of efficacy. Second, despite randomization, baseline imbalance was present in several functional measures. These imbalances could influence unadjusted longitudinal comparisons and therefore supported the decision to emphasize the baseline-adjusted mITT analysis. Third, one randomized participant withdrew before intervention and did not undergo baseline assessment, and some post-baseline outcome data were incomplete. Missing outcome data were not imputed; although the mixed-effects model used all available observations, missing data may still have introduced uncertainty. Fourth, the exploratory per-protocol analysis included a smaller population after excluding participants with eligibility violations. Although this analysis provides supportive information, it was not adjusted for baseline imbalance; therefore, nominal findings from the per-protocol repeated-measures analysis of variance (RM-ANOVA) should not be interpreted as confirmatory. Fifth, multiple outcome testing further limits interpretation of nominal uncorrected findings, as no group × time interaction remained robust after FDR correction. Sixth, K-MBI could not be estimated in the baseline-adjusted model because post-baseline values showed insufficient variability, limiting interpretation of activities-of-daily-living outcomes.

Seventh, complete participant blinding was difficult because of the perceptible nature of sensory stimulation, and therapists could not be blinded to intervention delivery. This may have introduced expectation-related or performance bias, although outcome assessment was blinded. Eighth, sensory thresholds could not be clearly determined in some participants because of hypesthesia on the hemiplegic side, which may have affected stimulation consistency. Finally, the study did not include direct neurophysiological assessments, such as measures of somatosensory function, corticospinal excitability, or sensorimotor integration. Although the FSR-based gait-phase detection algorithm was technically validated against a wearable IMU-based motion analysis system before application of the gait-synchronized stimulation protocol, validation was performed in a healthy adult rather than in all stroke participants. In addition, a laboratory-based three-dimensional optical motion capture system was not used as the reference standard, sensitivity and specificity were not calculated, and gait-phase detection accuracy was not continuously recorded during all intervention sessions. Therefore, the available validation data support the technical feasibility of the synchronization algorithm but do not establish phase-detection accuracy under all training conditions. Mechanistic interpretations regarding sensory input and sensorimotor integration remain speculative.

### 4.6 Future Directions

Future studies should evaluate these preliminary findings in larger, adequately powered, multicenter randomized controlled trials. Stratified randomization or prespecified covariate-adjusted designs may help reduce the influence of baseline functional imbalance and improve estimation of intervention-related effects. Future protocols should also consider intervention duration, training dose, and follow-up timing, as dose, intensity, and recovery trajectory are important design considerations in stroke rehabilitation research [33,34,35].

Further studies should also examine stimulation parameters, including intensity, timing, muscle targets, and phase-specific delivery algorithms. Direct neurophysiological assessments may help clarify whether and how different temporal profiles of peripheral sensory input influence somatosensory responsiveness, sensorimotor integration, or motor adaptation during post-stroke gait rehabilitation. Future studies should incorporate participant-specific validation of gait-phase detection, ideally using laboratory-based motion capture or synchronized wearable reference systems, and should record detection accuracy, missed events, false detections, and phase delay during intervention sessions. Individualized assessment of sensory responsiveness may also improve protocol consistency and support the development of more targeted sensory stimulation-based gait-training interventions.

## 5. Conclusions

In this pilot randomized trial, continuous and gait-synchronized TENS combined with standardized gait training were feasible and well tolerated in individuals with chronic stroke. The adjusted mITT analysis did not provide robust evidence of differential clinical effects among the stimulation-timing groups after correction for multiple outcome testing. These findings support the feasibility and safety of incorporating peripheral sensory stimulation into supervised gait training and suggest that the temporal profile of sensory input may warrant further investigation. Larger, adequately powered trials are needed to confirm whether continuous or gait-synchronized TENS provides clinically meaningful benefits during post-stroke gait rehabilitation.

## Data Availability

The datasets used and analyzed during the current study are available from the corresponding author on reasonable request.

## Abbreviations

TENS, transcutaneous electrical nerve stimulation; FAC, Functional Ambulation Category; CS, continuous stimulation; GS, gait-synchronized stimulation; FSR, force-sensing resistor; IMU, inertial measurement unit; 10MWT, 10-meter walk test; TUG, Timed Up and Go; BBS, Berg Balance Scale; MMT, manual muscle testing; K-MBI, Korean version of the Modified Barthel Index; RM-ANOVA, repeated-measures analysis of variance; mITT, modified intention-to-treat; PP, per-protocol; FDR, false discovery rate; CI, confidence interval; EQ-5D-3L, EuroQol-5 Dimension-3 Level.

## References

[1] Peurala SH, Pitkänen K, Sivenius J, Tarkka IM (2002). Cutaneous electrical stimulation may enhance sensorimotor recovery in chronic stroke. Clinical Rehabilitation.

[2] Park SH, Dee W, Keefer R, Roth EJ, Rymer WZ, Wu M (2023). Enhanced phasic sensory afferents paired with controlled constraint force improve weight shift toward the paretic side in individuals post-stroke. Journal of Stroke and Cerebrovascular Diseases: the Official Journal of National Stroke Association.

[3] Vecchio M, Chiaramonte R, De Sire A, Buccheri E, Finocchiaro P, Scaturro D (2024). Do proprioceptive training strategies with dual-task exercises positively influence gait parameters in chronic stroke? A systematic review. Journal of Rehabilitation Medicine.

[4] Tyson SF, Sadeghi-Demneh E, Nester CJ (2013). The effects of transcutaneous electrical nerve stimulation on strength, proprioception, balance and mobility in people with stroke: a randomized controlled cross-over trial. Clinical Rehabilitation.

[5] Park J, Seo D, Choi W, Lee S (2014). The effects of exercise with TENS on spasticity, balance, and gait in patients with chronic stroke: a randomized controlled trial. Medical Science Monitor: International Medical Journal of Experimental and Clinical Research.

[6] Cho JE, Shin JH, Kim H (2022). Does electrical stimulation synchronized with ankle movements better improve ankle proprioception and gait kinematics in chronic stroke? A randomized controlled study. NeuroRehabilitation.

[7] Kwong PW, Ng GY, Chung RC, Ng SS (2018). Transcutaneous electrical nerve stimulation improves walking capacity and reduces spasticity in stroke survivors: a systematic review and meta-analysis. Clinical Rehabilitation.

[8] Kwong PWH, Ng GYF, Chung RCK, Ng SSM (2018). Bilateral Transcutaneous Electrical Nerve Stimulation Improves Lower-Limb Motor Function in Subjects With Chronic Stroke: A Randomized Controlled Trial. Journal of the American Heart Association.

[9] Chastan N, Westby GWM, du Montcel ST, Do MC, Chong RK, Agid Y (2010). Influence of sensory inputs and motor demands on the control of the centre of mass velocity during gait initiation in humans. Neuroscience Letters.

[10] Huang Y, Tang Y, Zhang Z, Shen L, Guo Z, Chen Z (2026). Novel lower limb robot-assisted gait phase stimulation enhances walking and balance in stroke patients: a pilot trial. Journal of NeuroEngineering and Rehabilitation.

[11] Prasanth H, Caban M, Keller U, Courtine G, Ijspeert A, Vallery H (2021). Wearable sensor-based real-time gait detection: a systematic review. Sensors (Basel).

[12] Reisman DS, Wityk R, Silver K, Bastian AJ (2007). Locomotor adaptation on a split-belt treadmill can improve walking symmetry post-stroke. Brain: a Journal of Neurology.

[13] Helm EE, Reisman DS (2015). The Split-Belt Walking Paradigm: Exploring Motor Learning and Spatiotemporal Asymmetry Poststroke. Physical Medicine and Rehabilitation Clinics of North America.

[14] Tyrell CM, Helm E, Reisman DS (2014). Learning the spatial features of a locomotor task is slowed after stroke. Journal of Neurophysiology.

[15] Leon AC, Davis LL, Kraemer HC (2011). The role and interpretation of pilot studies in clinical research. Journal of Psychiatric Research.

[16] Julious SA (2005). Sample size of 12 per group rule of thumb for a pilot study. Pharmaceutical Statistics.

[17] Whitehead AL, Julious SA, Cooper CL, Campbell MJ (2016). Estimating the sample size for a pilot randomised trial to minimise the overall trial sample size for the external pilot and main trial for a continuous outcome variable. Statistical Methods in Medical Research.

[18] Park JS, Lee CM, Koo SM, Kim CH (2020). Gait phase detection using force sensing resistors. IEEE Sensors Journal.

[19] Diao Y, Niu X, Huang J, You C, Lin X, Pan J (2025). Superior efficacy of 100-Hz transcutaneous electrical nerve stimulation in reducing post-stroke spasticity: a systematic review and meta-analysis. Journal of Neuroengineering and Rehabilitation.

[20] Sullivan JE, Crowner BE, Kluding PM, Nichols D, Rose DK, Yoshida R (2013). Outcome measures for individuals with stroke: process and recommendations from the American Physical Therapy Association neurology section task force. Physical Therapy.

[21] Chan PP, Si Tou JI, Tse MM, Ng SS (2017). Reliability and Validity of the Timed Up and Go Test With a Motor Task in People With Chronic Stroke. Archives of Physical Medicine and Rehabilitation.

[22] Blum L, Korner-Bitensky N (2008). Usefulness of the Berg Balance Scale in stroke rehabilitation: a systematic review. Physical Therapy.

[23] Rensink M, Schuurmans M, Lindeman E, Hafsteinsdóttir T (2009). Task-oriented training in rehabilitation after stroke: systematic review. Journal of Advanced Nursing.

[24] Chen X, Liu F, Yan Z, Cheng S, Liu X, Li H (2018). Therapeutic effects of sensory input training on motor function rehabilitation after stroke. Medicine.

[25] Kleim JA, Jones TA (2008). Principles of experience-dependent neural plasticity: implications for rehabilitation after brain damage. Journal of Speech, Language, and Hearing Research: JSLHR.

[26] Maier M, Ballester BR, Verschure PFMJ (2019). Principles of Neurorehabilitation After Stroke Based on Motor Learning and Brain Plasticity Mechanisms. Frontiers in Systems Neuroscience.

[27] Kuo AD, Donelan JM (2010). Dynamic principles of gait and their clinical implications. Physical Therapy.

[28] Koganemaru S, Kitatani R, Fukushima-Maeda A, Mikami Y, Okita Y, Matsuhashi M (2019). Gait-Synchronized Rhythmic Brain Stimulation Improves Poststroke Gait Disturbance: A Pilot Study. Stroke.

[29] Kim HM, Na JM, Jo HS, Kim KH, Song MK, Park HK (2024). Feasibility of simultaneous anodal transcranial direct current stimulation during gait training in chronic stroke patients: a randomized double-blind pilot clinical trial. Journal of Integrative Neuroscience.

[30] Hafsteinsdóttir TB, Rensink M, Schuurmans M (2014). Clinimetric properties of the Timed Up and Go Test for patients with stroke: a systematic review. Topics in Stroke Rehabilitation.

[31] Aries AM, Downing P, Sim J, Hunter SM (2022). Effectiveness of Somatosensory Stimulation for the Lower Limb and Foot to Improve Balance and Gait after Stroke: A Systematic Review. Brain Sciences.

[32] Lewek MD, Feasel J, Wentz E, Brooks FP, Whitton MC (2012). Use of visual and proprioceptive feedback to improve gait speed and spatiotemporal symmetry following chronic stroke: a case series. Physical Therapy.

[33] Lohse KR, Lang CE, Boyd LA (2014). Is more better? Using metadata to explore dose-response relationships in stroke rehabilitation. Stroke.

[34] Kwakkel G, Kollen B, Lindeman E (2004). Understanding the pattern of functional recovery after stroke: facts and theories. Restorative Neurology and Neuroscience.

[35] Ballester BR, Ward NS, Brander F, Maier M, Kelly K, Verschure PFMJ (2022). Relationship between intensity and recovery in post-stroke rehabilitation: a retrospective analysis. Journal of Neurology, Neurosurgery, and Psychiatry.

