# Supplementary material for: Comparison of Continuous and Gait-synchronized Transcutaneous Electrical Nerve Stimulation During Gait Training in Chronic Stroke: A Pilot Randomized Trial
Source: Rev Neurol. 2026 Aug 25;81(8):53766. doi: 10.31083/RN53766 (PMC13541420; doi:10.31083/RN53766)
Supplement: Supplementary file 1 [file 1576-6578-81-8-53766-s1.zip › Supplementary Material/Supplementary Material-V2.pdf]

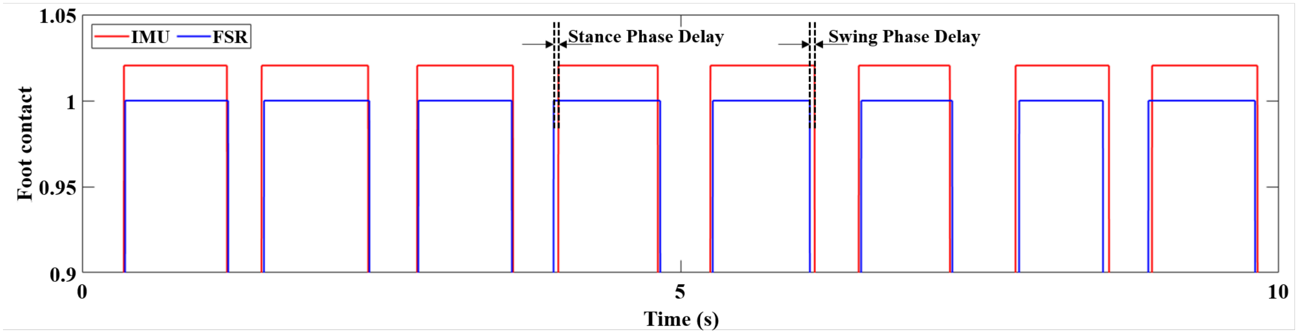

Supplementary Fig. 1. Representative validation of gait-phase detection using the FSR-based insole system. Representative left foot-contact data obtained during overground walking. The red line indicates foot contact detected using the wearable IMU-based motion analysis system, and the blue line indicates foot contact detected using the FSR-based insole sensors. In a validation test involving 200 overground walking steps from a healthy young adult, the FSR-based insole system detected 198 of 200 foot-contact events, corresponding to a foot-contact event detection rate of 99% and missed-event rate of 1%. The mean phase delay was 25.62 ± 14.06 ms for stance-phase detection and 28.20 ± 12.89 ms for swing-phase detection. Abbreviations: FSR, force-sensing resistor; IMU, inertial measurement unit.

Supplementary Table 1. Baseline-adjusted linear mixed-effects model results for manual muscle testing in the modified intention-to-treat population.

MMT	Model term	F	df1	df2	*P*-value	FDR-adjusted *P*-value
Ipsilesional hip	Group	4.39	2	24.24	0.024	-
	Time	7.778	1	22.14	0.011	-
	Group × Time	0.087	2	22.08	0.917	0.917
Ipsilesional knee	Group	5.58	2	23.94	0.010	-
	Time	8.07	1	22.05	0.009	-
	Group × Time	0.087	2	22.01	0.917	0.917
Ipsilesional ankle	Group	1.95	2	24.47	0.164	-
	Time	9.427	1	22.23	0.006	-
	Group × Time	0.378	2	22.19	0.690	0.917
Contralesional hip	Group	2.442	2	42	0.099	-
	Time	6.384	1	42	0.015	-
	Group × Time	3.319	2	42	0.046	0.092
Contralesional knee	Group	2.442	2	42	0.099	-
	Time	6.384	1	42	0.015	-
	Group × Time	3.319	2	42	0.046	0.092
Contralesional ankle	Group	2.442	2	42	0.099	-
	Time	6.384	1	42	0.015	-
	Group × Time	3.319	2	42	0.046	0.092

Values are results from baseline-adjusted linear mixed-effects models fitted separately for each manual muscle testing outcome. Post-intervention and follow-up values were modeled as repeated outcomes, with the corresponding baseline value included as a covariate. Fixed effects included group, time, group × time interaction, and baseline value, with participant ID included as a random intercept. Manual muscle testing grades were converted to numeric scores for exploratory analysis; full grades were coded from 0 to 5, and minus and plus modifiers were coded as −0.33 and +0.33 from the corresponding full grade, respectively. FDR-adjusted p-values were calculated using the Benjamini–Hochberg procedure for the group × time interaction terms across the six manual muscle testing outcomes. Because manual muscle testing is ordinal, these findings should be interpreted descriptively. Abbreviations: MMT, manual muscle testing; df, degrees of freedom; FDR, false discovery rate.

Supplementary Table 2. Baseline-adjusted pairwise comparisons between groups at each post-baseline time point in the modified intention-to-treat population.

Variables	Time	Comparison	Adjusted mean difference	95% CI	Adjusted *P*-value
Comfortable speed (m/s)	T1	CS vs GS	0.042	-0.108 to 0.192	0.775
	T1	CS vs Sham	-0.009	-0.158 to 0.141	0.989
	T1	GS vs Sham	-0.05	-0.174 to 0.074	0.585
	T2	CS vs GS	0.034	-0.117 to 0.185	0.844
	T2	CS vs Sham	0.048	-0.109 to 0.204	0.739
	T2	GS vs Sham	0.013	-0.117 to 0.143	0.965
Maximal speed (m/s)	T1	CS vs GS	0.045	-0.081 to 0.171	0.663
	T1	CS vs Sham	0.005	-0.122 to 0.132	0.996
	T1	GS vs Sham	-0.04	-0.150 to 0.069	0.647
	T2	CS vs GS	0.126	-0.001 to 0.254	0.053
	T2	CS vs Sham	0.081	-0.055 to 0.218	0.326
	T2	GS vs Sham	-0.045	-0.162 to 0.073	0.626
Timed Up and Go test (s)	T1	CS vs GS	-1.214	-3.841 to 1.412	0.500
	T1	CS vs Sham	1.428	-1.416 to 4.271	0.442
	T1	GS vs Sham	2.642	0.035 to 5.249	0.046
	T2	CS vs GS	-1.128	-3.783 to 1.526	0.556
	T2	CS vs Sham	0.214	-2.810 to 3.239	0.984
	T2	GS vs Sham	1.343	-1.406 to 4.092	0.466
Berg Balance Scale (points)	T1	CS vs GS	1.336	-3.437 to 6.109	0.774
	T1	CS vs Sham	-0.482	-5.180 to 4.216	0.966
	T1	GS vs Sham	-1.818	-5.489 to 1.854	0.457
	T2	CS vs GS	-1.687	-6.548 to 3.175	0.676
	T2	CS vs Sham	0.676	-4.421 to 5.773	0.944

	T2	GS vs Sham	2.363	-1.658 to 6.384	0.338
Functional Ambulatory Category (level)	T1	CS vs GS	0.013	-0.454 to 0.480	0.998
	T1	CS vs Sham	-0.236	-0.692 to 0.219	0.428
	T1	GS vs Sham	-0.249	-0.691 to 0.192	0.367
	T2	CS vs GS	0.012	-0.467 to 0.491	0.998
	T2	CS vs Sham	0.298	-0.230 to 0.826	0.367
	T2	GS vs Sham	0.286	-0.220 to 0.792	0.367
EQ-5D-3L (sum score)	T1	CS vs GS	-0.663	-2.002 to 0.677	0.460
	T1	CS vs Sham	-1.198	-2.498 to 0.102	0.076
	T1	GS vs Sham	-0.535	-1.834 to 0.764	0.582
	T2	CS vs GS	-0.07	-1.464 to 1.325	0.992
	T2	CS vs Sham	-1.992	-3.498 to -0.486	0.007
	T2	GS vs Sham	-1.922	-3.385 to -0.460	0.007

Pairwise comparisons were performed using estimated marginal means from the baseline-adjusted linear mixed-effects models. Comparisons between groups were conducted separately at each post-baseline time point, and p-values were adjusted using Tukey's method. Adjusted mean differences are presented with 95% confidence intervals. Pairwise comparisons for K-MBI are not presented because K-MBI showed insufficient post-baseline variability for reliable estimation in the baseline-adjusted mixed-effects model. Higher EQ-5D-3L sum scores indicate more problems. Abbreviations: CS, continuous stimulation; GS, gait-synchronized stimulation; CI, confidence interval; T1, post-intervention; T2, follow-up; K-MBI, Korean version of the Modified Barthel Index; EQ-5D-3L, EuroQol-5 Dimension-3 Level.

Supplementary Table 3. Exploratory per-protocol repeated-measures ANOVA results for primary and secondary outcomes.

	Group	T0	T1	T2	Time effect[a]	Group × Time effect[a]	ηp^2 (partial eta squared)
Primary outcomes							
10-meter walk test, (m/s)							
Comfortable speed	CS	0.97 ± 0.16	0.96 ± 0.15	1.04 ± 0.14	0.239		
	GS	0.65 ± 0.17	0.63 ± 0.20	0.71 ± 0.19	0.139	0.005*	0.228
	Sham	0.63 ± 0.27	0.66 ± 0.25	0.68 ± 0.26	0.133		
Maximal speed	CS	1.16 ± 0.22	1.16 ± 0.20	1.26 ± 0.19	0.067		
	GS	0.82 ± 0.20	0.81 ± 0.22	0.84 ± 0.19	0.494	0.009*	0.261
	Sham	0.75 ± 0.36	0.80 ± 0.31	0.83 ± 0.32	0.097		
Secondary outcomes							
Timed Up and Go test (s)	CS	7.18 ± 1.24	7.27 ± 1.40	6.63 ± 1.01	0.019*		
	GS	10.23 ± 2.07	10.56 ± 3.13	10.04 ± 3.33	0.662	0.026*	0.130
	Sham	13.53 ± 6.47	11.46 ± 4.03	11.80 ± 5.28	0.156		
Berg Balance Scale (points)	CS	45.13 ± 5.41	45.50 ± 5.18	47.29 ± 5.56	0.223		
	GS	32.50 ± 5.09	33.83 ± 4.49	37.67 ± 3.39	0.006*	0.001*	0.421
	Sham	31.14 ± 7.67	34.71 ± 6.42	35.50 ± 5.74	0.068		
Functional Ambulatory Category (level)	CS	4.50 ± 0.53	4.50 ± 0.53	4.71 ± 0.49	0.154		
	GS	3.83 ± 0.41	3.83 ± 0.41	4.17 ± 0.41	0.175	0.038*	0.197
	Sham	3.71 ± 0.76	4.00 ± 0.82	4.00 ± 0.82	-		
Korean version of the Modified Barthel Index (points)	CS	87.88 ± 5.67	87.88 ± 5.67	87.57 ± 6.05	-		
	GS	84.17 ± 2.64	84.17 ± 2.64	84.17 ± 2.64	-	0.183	-
	Sham	79.43 ± 12.84	79.43 ± 12.84	82.75 ± 13.82	-		
EQ-5D-3L (sum score)	CS	7.25 ± 2.25	6.88 ± 1.73	6.86 ± 1.57	0.341		
	GS	8.33 ± 1.97	8.17 ± 1.60	7.83 ± 2.23	0.591	0.090	0.030
	Sham	9.00 ± 1.41	9.00 ± 1.63	10.00 ± 0.00	0.194		

These exploratory per-protocol analyses were not adjusted for baseline imbalance and should not be interpreted as confirmatory. Values are presented as

mean ± standard deviation. Time and group × time interaction effects are presented as p-values obtained using repeated-measures ANOVA, with Greenhouse–Geisser correction where appropriate. *$p < 0.05$. – Analysis was not performed due to insufficient variability. Higher EQ-5D-3L sum scores indicate more problems. Abbreviations: CS, continuous stimulation; GS, gait-synchronized stimulation; TUG, Timed Up and Go; BBS, Berg Balance Scale; FAC, Functional Ambulatory Category; K-MBI, Korean version of the Modified Barthel Index; EQ-5D-3L, EuroQol-5 Dimension-3 Level.
